# Herbal Medicine in Breast Cancer Therapy: Mechanisms, Evidence, and Future Perspectives

**DOI:** 10.3390/cimb47050362

**Published:** 2025-05-15

**Authors:** Hsien-Chang Wu, Chung-Che Tsai, Po-Chih Hsu, Chan-Yen Kuo

**Affiliations:** 1 School of Post-baccalaureate Chinese Medicine, Tzu Chi University, Hualien 970, Taiwan; xuang@tzuchi.com.tw; 2 Department of Chinese Medicine, Taipei Tzu Chi Hospital, The Buddhist Tzu Chi Medical Foundation, No. 289, Jianguo Rd., Xindian Dist, New Taipei City 231, Taiwan; 3 Department of Research, Taipei Tzu Chi Hospital, The Buddhist Tzu Chi Medical Foundation, New Taipei City 231, Taiwan; chungche.tsai@gmail.com; 4 Department of Nursing, Cardinal Tien College of Healthcare and Management, New Taipei City 231, Taiwan; 5 Department of Dentistry, Taipei Tzu Chi Hospital, Buddhist Tzu Chi Medical Foundation, New Taipei City 231, Taiwan; 6 Institute of Oral Medicine and Materials, College of Medicine, Tzu Chi University, Hualien 970, Taiwan

**Keywords:** breast cancer, Chinese herbal medicine, therapy

## Abstract

Breast cancer remains a leading global cause of cancer-related mortality among women, requiring the development of safer and more effective therapeutic strategies. Herbal medicines have gained increasing attention as complementary approaches due to their multi-targeted actions, more limited toxicities, and the potential ability to overcome resistance associated with conventional treatments. This review highlights the antitumor properties and underlying mechanisms of several well-studied herbal compounds, including curcumin, resveratrol, epigallocatechin gallate, withaferin A, thymoquinone, baicalin, berberine, *Oldenlandia diffusa*, and *Salvia miltiorrhiza*. These phytochemicals exert antitumor effects by inducing apoptosis, inhibiting cell proliferation and metastasis, modulating immune responses, and sensitizing tumor cells to chemotherapy and radiotherapy. Furthermore, many of these agents regulate key signaling pathways, such as nuclear factor kappa-light-chain-enhancer of activated B cells, phosphatidylinositol 3-kinase/AKT, p53, signal transducer and activator of transcription 3, and extracellular signal-regulated kinases 1/2, and the tumor microenvironment. Despite promising preclinical and early clinical evidence, challenges remain regarding the bioavailability, standardization, and large-scale clinical validation of these phytochemicals. This review underscores the therapeutic potential of herbal medicines in breast cancer treatment and advocates for further research to facilitate their integration into evidence-based oncology practice.

## 1. Introduction

Breast cancer is the most common form of cancer and the leading cause of cancer-related deaths among women worldwide [1]. Despite significant advances in diagnostic and therapeutic approaches, including surgery, chemotherapy, radiation, hormone therapy, and targeted agents, challenges, such as treatment resistance, recurrence, and adverse effects, persist [2]. Therefore, many patients seek complementary and alternative therapies that can enhance treatment efficacy, alleviate side effects, and improve the overall quality of life [3].

Herbal medicine, an integral component of traditional medical systems, including Traditional Chinese medicine and Ayurveda, is increasingly recognized for its potential utility in cancer therapy [4,5]. Herbal medicines, derived from various plant sources, contain bioactive compounds that exert diverse pharmacologic effects, including antitumor, anti-inflammatory, antioxidant, and immunomodulatory effects, and apoptosis [6,7].

In breast cancer, numerous herbal extracts and phytochemicals have been shown to exert promising effects in inhibiting tumor proliferation, metastasis, angiogenesis, and drug resistance in preclinical studies [8,9]. Some herbal compounds exert synergistic effects with conventional chemotherapy and hormone therapy, potentially enhancing their efficacy and minimizing their toxicity [10]. Additionally, certain herbal formulations are utilized to support immune function and reduce chemotherapy-induced side effects, such as fatigue, nausea, myelosuppression, and neuropathy [11,12].

Despite encouraging findings, the integration of herbal medicines into mainstream oncology remains limited due to several factors, including variability in formulations, lack of standardized dosing, and insufficient high-quality clinical trials. Nevertheless, the continuation of research that combines modern pharmacologic techniques with traditional knowledge offers a compelling approach to develop safer, more effective, and personalized therapeutic strategies for breast cancer.

This review aims to critically evaluate the therapeutic potential and underlying mechanisms of selected traditional herbal medicines—specifically, curcumin, *Scutellaria baicalensis*, *Oldenlandia diffusa*, and *Salvia miltiorrhiza*—in the treatment of breast cancer. By integrating findings from both preclinical and clinical studies, we seek to provide a comprehensive overview of how these herbal compounds may contribute to breast cancer therapy.

## 2. Traditional Herbal Medicines in Breast Cancer Treatment: Mechanisms and Therapeutic Potential of Curcumin, *Scutellaria baicalensis*, *Oldenlandia diffusa*, and *Salvia miltiorrhiza*

Curcumin is the main polyphenolic compound extracted from *Curcuma longa* (turmeric), a plant known for its distinctive orange/yellow pigmentation [13]. Multiple preclinical studies in breast cancer have demonstrated that curcumin effectively inhibits tumor cell proliferation, induces apoptosis, and suppresses metastasis, with supportive evidence also emerging from clinical studies. These effects are attributed to the modulation of multiple molecular pathways involved in cancer progression (Table 1) [14,15]. Studies indicate that curcumin might suppress breast cancer cell proliferation by downregulating flap endonuclease 1 expression via nuclear factor erythroid 2-related factor 2 (NRF2) signaling [16]. In breast cancer-bearing mice, intravenous curcumin administration significantly suppresses tumor growth and inhibited metastasis [17]. Either alone or in combination with tamoxifen, curcumin exhibits therapeutic potential to overcome endocrine resistance in breast cancer, mediated through the inhibition of tumor cell proliferation, the promotion of apoptosis, and the involvement of multiple survival- and resistance-related signaling pathways [18]. In clinical trials, curcumin in combination with docetaxel was shown to inhibit the progression of breast cancer and reduce the levels of tumor markers, similarly to the effects observed with each compound individually [19,20,21]. Combination treatment with curcumin and paclitaxel for 12 weeks demonstrated superior efficacy, including overall response rate and physical performance, compared to the paclitaxel–placebo regimen. Intravenous curcumin administration was well tolerated, with no significant safety concerns or adverse effects on the quality of life; curcumin also appeared to aid in alleviating fatigue [15]. However, the main challenges in using curcumin for the treatment of breast cancer are low bioavailability, rapid metabolism, and limited water solubility [22]. Nanoparticle-based delivery systems, such as polymeric nanoparticles, carbon nanotubes, and liposomes, have been developed to enhance the bioavailability and therapeutic efficacy of curcumin [23]. Encapsulating curcumin in liposomes has been shown to improve its stability and bioavailability. For instance, co-delivery liposomes containing curcumin and docetaxel (CUR-DTX-L) demonstrated enhanced antitumor efficacy in MCF-7 breast cancer models, with improved pharmacokinetic parameters such as increased half-life and mean residence time compared to free drugs [24]. On the other hand, curcumin-loaded solid lipid nanoparticles (SLNs) have exhibited stronger cytotoxicity against breast cancer cells and higher cellular uptake efficiency [25]. These nanoparticles also induced higher apoptosis rates compared to free curcumin, suggesting their potential as effective chemotherapeutic formulations [26]. Modifications such as RGD peptide-mediated liposomes have been employed to enhance the targeted delivery of curcumin to breast cancer cells. These systems have shown significant cytotoxic effects and induced higher apoptosis rates in MCF-7 cells compared to non-targeted formulations [27,28]. For pharmacokinetic improvements on curcumin, the CUR-DTX-L formulation increased the plasma concentration–time curve, mean residence time, and biological half-life of curcumin compared to free drugs, indicating prolonged circulation and sustained release [29]. SLNs have been reported to modulate release kinetics and improve blood circulation time, thereby increasing the overall therapeutic efficacy of curcumin [30].

In summary, treatment with curcumin, either alone or in combination with other chemotherapeutic agents, exhibits substantial therapeutic potential in breast cancer through its multifaceted biological activities, including the inhibition of cell proliferation, induction of apoptosis, suppression of metastasis, and reversal of endocrine resistance. These effects of curcumin are mediated through the modulation of key molecular pathways, such as NRF2/flap endonuclease 1 signaling. Both preclinical and clinical studies demonstrate the efficacy of curcumin, especially in combination with chemotherapeutic agents, such as tamoxifen, docetaxel, and paclitaxel. Although the intravenous administration of curcumin has shown promising results with minimal adverse effects, its clinical applicability is limited by poor bioavailability and rapid metabolism. Advanced drug delivery systems, particularly nanoparticle-based formulations, offer a promising strategy to overcome these limitations and enhance the therapeutic utility of curcumin in breast cancer. Table 2 provides a concise overview of the current clinical and preclinical evidence supporting the potential use of these herbal compounds in breast cancer treatment. It highlights the need for further clinical trials to validate the efficacy and safety of *Scutellaria baicalensis* and *Oldenlandia diffusa*, while acknowledging the promising results observed with curcumin and *Salvia miltiorrhiza*.

Resveratrol (trans-3,4′,5-trihydroxystilbene), a natural polyphenol found in grapes, peanuts, cocoa, berries, and red wine, is recognized for its diverse biologic activities in breast cancer, including antioxidant, cardioprotective, neuroprotective, anti-inflammatory, and antitumor effects [35,36]. Kim et al. reported that resveratrol attenuated breast cancer cell invasion by inactivating the RhoA/Lats1/YAP signaling pathway. Specifically, resveratrol inactivated RhoA, leading to the activation of Lats1 kinase, which in turn phosphorylated and inactivated YAP, a key transcriptional coactivator involved in cell proliferation and invasion. The inactivation of this cascade led to the suppression of the expression levels of YAP target genes, with a consequent reduction in the invasive behavior of breast cancer cells [37]. When used in combination with chemotherapeutic agents, such as docetaxel, paclitaxel, cisplatin, and doxorubicin, resveratrol enhances antitumor activity in breast cancer cells [38,39,40]. However, the clinical use of resveratrol is limited by its poor water solubility, which has led to the development of nano-based drug delivery systems to improve its bioavailability [41]. Resveratrol was demonstrated to inhibit cancer-associated fibroblast-induced proliferation, migration, invasion, and stemness of breast cancer cells. Resveratrol downregulated key oncogenic proteins (Cyclin D1, c-MYC, matrix metalloproteinase [MMP]-2, MMP-9), suppressed SRY-box 2, and blocked AKT and inhibited signal transducer and activator of transcription 3 signaling. These findings suggest that resveratrol disrupts the interactions between tumor cells and cancer-associated fibroblasts and imply that targeting the tumor microenvironment might underline the therapeutic potential of resveratrol [42].

Liang et al. demonstrated that resveratrol significantly inhibited the proliferation of TNBC cells by downregulating DNA polymerase delta catalytic subunit (*POLD1*), a gene associated with DNA replication and repair, leading to the induction of apoptosis, evidenced by the increased expression of apoptotic markers, such as cleaved poly(ADP-ribose) polymerase 1 and cleaved caspase 3 [33]. A similar study reported that resveratrol suppressed estrogen-induced breast carcinogenesis by activating the NRF2-mediated protective signaling pathway [43]. However, contradictory studies indicated that resveratrol promoted tumor growth by shortening tumor latency and increasing tumor number; mechanistic experiments revealed that resveratrol acted as a proteasome inhibitor, leading to the accumulation of Δ16HER2, the reduction in the expression of estrogen receptor alpha (ERα), and the activation of mTORC1 signaling, thereby promoting cancer cell proliferation [44]. Altogether, accumulating evidence supports the promising antitumor potential of resveratrol in breast cancer, mediated through multiple molecular mechanisms. Despite these benefits, conflicting evidence highlights the potential tumor-promoting effects of resveratrol in specific molecular subtypes, such as HER2^+^/ERα^+^ breast cancer, where resveratrol may act as a proteasome inhibitor and activate pathways that promote tumor cell proliferation. Therefore, despite its therapeutic promise, the utility of resveratrol in clinical settings requires further investigation, especially regarding cancer subtype-specific responses and optimized delivery strategies.

Epigallocatechin gallate (EGCG), the major catechin in green tea, possesses potent antioxidant, anti-inflammatory, cardioprotective, and antitumor properties [45]. In one study, EGCG was shown to exert notable antitumor effects against Michigan Cancer Foundation (MCF)-7 breast cancer cells in both in vitro and in vivo models. Specifically, EGCG induced apoptosis, disrupted cell cycle progression by blocking the G2/M transition, and modulated the expression of key apoptotic markers by downregulating miR-25; the restoration of miR-25 reversed these effects, confirming its role in EGCG-mediated apoptosis in these models. In vivo, EGCG suppressed tumor growth and promoted apoptotic signaling, evidenced by a decrease in Ki-67 and an increase in poly(ADP-ribose) polymerase 1 expression [46]. Epidemiologic studies indicate that EGCG might be protective against hormone-related cancers, particularly breast and prostate cancers [47]. Interestingly, EGCG suppressed proliferation, invasion, lymphangiogenesis, and stem-like cell properties in inflammatory breast cancer cells, particularly by downregulating vascular endothelial growth factor (VEGF)-D and reducing the growth of aldehyde dehydrogenase-positive stem-like populations. In vivo, EGCG was also shown to significantly impair tumor growth and lymphatic vessel formation, highlighting its potential in reducing recurrence and improving outcomes [48]. In MDA-MB-231 cells, combination treatment with EGCG and curcumin induced significant cell cycle arrest in the G2/M phase and reduced cell viability, while leading to a notable reduction in tumor volume and a marked suppression in VEGF receptor 1 expression, implicating the downregulation of this receptor a key mechanism underlying the efficacy of the combination treatment in vivo [49]. Similar studies reported that combination treatment with curcumin and EGCG inhibited the cancer stem cell characteristics, including the ability to form tumor spheres and CD44^+^ cell populations, in breast cancer. Functionally, the combination of curcumin with EGCG inhibited the phosphorylation of signal transducer and activator of transcription 3 and its interaction with nuclear factor kappa-light-chain-enhancer of activated B cells (NF-κB), two key molecules supporting the survival and self-renewal of cancer stem cells [50]. One randomized clinical trial demonstrated that the prophylactic use of a topical EGCG solution significantly reduced the incidence and severity of radiation-induced dermatitis in patients with breast cancer undergoing adjuvant radiotherapy [51]. Next-generation sequencing identified over 1500 known and novel miRNAs, with EGCG treatment significantly modulating the expression of 873 known and 47 novel miRNAs. Bioinformatic analysis (KEGG and PANTHER) confirmed that these miRNAs were involved in key cancer-related pathways in MDA-MB-231 cells, a breast cancer cell line [52]. Altogether, these findings support the role of EGCG as a promising multifunctional agent for breast cancer prevention, treatment, and supportive care, warranting further investigation of its utility in clinical settings.

Withaferin A (WA), a bioactive compound isolated from *Withania somnifera* (Ashwagandha), has significant antitumor potential, which has been reported in numerous in vitro and in vivo studies [53]. In breast cancer cells, WA was shown to inhibit aerobic glycolysis by downregulating key glycolytic enzymes, including glucose transporter 1, hexokinase 2, and pyruvate kinase muscle isozyme 2, and by targeting the c-MYC pathway. WA was reported to reduce glucose uptake, lactate production, and ATP generation, thereby leading to decreased cell viability and tumor-forming capacity [54]. In addition, WA was reported to partially reverse epithelial–mesenchymal transition (EMT) induced by tumor necrosis factor α and transforming growth factor β in non-tumorigenic MCF-10A cells and to modulate EMT markers, such as E-cadherin and vimentin, in breast cancer cell lines. Furthermore, one study reported that WA significantly reduced vimentin expression in both xenograft and transgenic mouse tumor models, associated with its antitumor effects [55]; the same study demonstrated that WA selectively induced reactive oxygen species (ROS) production and apoptosis in MDA-MB-231 and MCF-7 breast cancer lines, while sparing normal mammary epithelial cells. The proapoptotic effects of WA were linked to the suppression of mitochondrial oxidative phosphorylation, specifically complex III activity, which was dependent on the presence of mitochondrial DNA and the activation of B-cell lymphoma 2 (BCL-2)-associated X protein (BAX)/BCL-2 antagonist/killer signaling [56]. Molecular docking and mutagenesis studies confirmed the critical residues involved in the binding of WA to TWIK-related acid-sensitive K^+^ channel 3, with WA exerting a dose-dependent, voltage-independent inhibition of TWIK-related acid-sensitive K^+^ channel 3 activity, which contributed to its cytotoxic effects in breast cancer cells [57]. In breast cancer cells, WA was shown to induce apoptosis by disrupting mitochondrial dynamics and inhibiting complex III of the electron transport chain through the regulation of mitochondrial fusion proteins, such as mitofusin 1 and 2 and optic atrophy protein 1. WA also suppressed the expression of dynamin-related protein 1, which regulates mitochondrial fission, leading to mitochondrial fragmentation and volume loss [58]. Another study provides mechanistic insights into the proapoptotic action of WA in human breast cancer cells, demonstrating that its antitumor effects are partially mediated through the modulation of the p53 and ERα pathways [59]. Interestingly, the results reveal a novel mechanism by which WA exerts its antitumor effects on breast cancer. WA was shown to activate the extracellular signal-regulated kinase (ERK)/ribosomal S6 kinase signaling cascade, inducing the transcription of death receptor 5 via the ETS-like transcription factor 1/C-EBP homologous protein pathway and sensitizing cancer cells to proapoptotic agents, such as celecoxib, etoposide, and TRAIL. In xenograft and MMTV-neu mouse models in vivo, WA was shown to effectively suppress breast tumor growth, in association with the activation of ERK/ribosomal S6 kinase signaling, the upregulation of death receptor 5, and the increased nuclear localization of ETS-like transcription factor 1 and C-EBP homologous protein [60]. Kim et al. reported that Notch2 functioned as a crucial regulator of tumor growth in TNBC and that WA was effective in restoring the tumor-suppressive effects of Notch2 [61]. Another study revealed that WA inhibited breast cancer growth by disrupting cellular energy homeostasis; WA was a potent lysosomal inhibitor and blocked the autophagic flux and lysosomal proteolysis, thereby impairing the recycling of metabolic substrates essential for the tricarboxylic acid cycle and oxidative phosphorylation [62]. Collectively, these findings underscore the potential of WA as a multi-targeted therapeutic agent in breast cancer, particularly in aggressive and therapy-resistant subtypes. The ability of WA to interfere with metabolism, mitochondrial function, survival signaling, and immune evasion highlights the need for further preclinical and clinical studies to determine its potential as a novel antitumor strategy.

In recent years, a growing number of studies have highlighted the diverse medicinal properties of thymoquinone, the primary bioactive compound found in *Nigella sativa* (black seed) [63]. Accumulating evidence demonstrates that thymoquinone targets multiple tumorigenic pathways to promote apoptosis, inhibit metastasis, and regulate key signaling molecules, such as p53, NF-κB, ERK1/2, and phosphatidylinositol 3-kinase [64]. Thymoquinone was shown to enhance the cytotoxic function of natural killer cells against MCF-7 cells by increasing the secretion of key cytolytic molecules, including perforin, granzyme B, and interferon α [65]. Woo et al. demonstrated that thymoquinone exerted potent antitumor activity in breast cancer cells by inducing ROS production, with the downstream activation of p38 phosphorylation, consequently leading to the inhibition of proliferation and the induction of apoptosis. Thymoquinone was shown to effectively downregulate antiapoptotic proteins, such as X-linked inhibitor of apoptosis, survivin, BCL-xL, and BCL-2, and to upregulate apoptotic markers in tumor tissues. In vivo, thymoquinone was demonstrated to suppress tumor growth, an effect further potentiated by doxorubicin, and to boost antioxidant enzyme levels in liver tissue, suggesting a protective role [66]. Conversely, thymoquinone holds significant potential for both the prevention and treatment of breast cancer, with one study providing the first evidence that its antitumor effects may, in part, be mediated through the modulation of the peroxisome proliferator-activated receptor γ pathway [67]. In triple-negative breast cancer, thymoquinone was found to exert an antimetastatic effect by downregulating the expression of C-X-C chemokine receptor type 4 through the inhibition of NF-κB signaling. In vivo, thymoquinone effectively reduced tumor growth, vascularity, and metastatic spread to the lungs, brain, and bones, while diminishing osteolytic lesions and the expression of metastatic markers [68]. Collectively, these findings support the potential of thymoquinone as a complementary or stand-alone therapeutic strategy in breast cancer.

*Scutellaria baicalensis* (Chinese skullcap), a perennial herb belonging to the mint family (*Lamiaceae*), is widely used in traditional medicine in East Asia [69]. *Scutellaria baicalensis* exerts anti-breast cancer effects by regulating transcription and modulating various kinases, ultimately leading to alterations in phosphorylation within cell signaling pathways (Table 3) [70]. Baicalin and baicalein, two bioactive flavonoids derived from *Scutellaria baicalensis*, have been shown to exert therapeutic effects in breast cancer [71]. Specifically, in highly aggressive MDA-MB-231 breast cancer cells, baicalin was reported to suppress metastasis by targeting β-catenin signaling and reversing the EMT [72]. In MCF-7 cells, combination treatment with baicalin and baicalein was shown to enhance apoptosis by activating caspases 9 and 3, downregulating BCL-2, and upregulating BAX and p53, mediated through the ERK/p38 mitogen-activated protein kinase (MAPK) pathway [73]. Park et al. reported that the *Scutellaria baicalensis* Georgi extract (SBGE) inhibited cell proliferation and induced apoptosis by downregulating BCL-2, upregulating BAX, activating caspases 3 and 9, and increasing ROS generation. Additionally, the apoptotic effects of the SBGE were mediated through the activation of the MAPK pathway, based on the reduction in SBGE-induced cell death observed with the inhibition of MAPK and c-Jun N-terminal kinase [74]. Another study demonstrated that the whole extract of *Scutellaria baicalensis*, i.e., SbE, did not exert a significant inhibitory effect in MCF-7 cells but that a specific fraction of SbE was chemopreventive [75]. In conclusion, *Scutellaria baicalensis* exhibits significant therapeutic potential in breast cancer, by regulating transcription factors and kinase activity, leading to alterations in key signaling pathways. The bioactive flavonoids baicalin and baicalein play crucial roles in inhibiting metastasis, inducing apoptosis, and modulating apoptotic proteins, such as BAX, BCL-2, and p53, via the ERK/p38 MAPK pathway. Furthermore, the SBGE suppresses proliferation and enhances apoptosis in breast cancer cells through the generation of ROS and the activation of the MAPK pathway. Although SbE may not exert a strong inhibitory effect in breast cancer cells, specific fractions of SbE exhibit promising chemopreventive properties, highlighting the need for further research to optimize its therapeutic potential.

Berberine overcomes hypoxia-induced chemoresistance in breast cancer by inhibiting the AMP-activated protein kinase/hypoxia-inducible factor 1α signaling pathway [76]. Kim et al. demonstrated that berberine effectively suppressed the 12-O-tetradecanoylphorbol-13-acetate (TPA)-induced expression of VEGF and fibronectin as well as the VEGF-induced fibronectin expression in breast cancer cells. These effects were mediated through the inhibition of the phosphatidylinositol 3-kinase/AKT signaling pathway. Given its ability to downregulate key angiogenic factors, berberine holds promise as a therapeutic agent targeting angiogenesis in breast cancer [77]. In HER2^+^ breast cancer cells, berberine was shown to effectively overcome lapatinib resistance by disrupting redox homeostasis. Specifically, berberine induced ROS-mediated apoptosis in lapatinib-resistant cells by downregulating the c-MYC/pro-NRF2 signaling and inhibiting glycogen synthase kinase 3β-mediated NRF2 stabilization [78]. Collectively, these findings underscore berberine’s potential as an adjunctive therapeutic targeting multiple resistance and survival pathways in breast cancer.

*Oldenlandia diffusa* (*Willd.*) *Roxb*., a plant widely distributed in the southern provinces of China, is commonly used in Traditional Chinese medicine [79]. *Oldenlandia diffusa* is notable for its strong antitumor and anti-inflammatory properties (Table 4) [80]. Specifically, the *Oldenlandia diffusa* extract was shown to inhibit cell proliferation and induced apoptosis by upregulating p53 expression via the ERα/SP1 signaling pathway. Additionally, we identified that ursolic and oleanolic acids isolated from the *Oldenlandia diffusa* extract were bioactive compounds responsible for these effects [81]. Chung et al. reported that *Oldenlandia diffusa* functioned as an antimetastatic agent by reducing the invasion capacity of MCF-7 breast cancer cells through the inhibition of p-ERK, p38, and NF-κB signaling and the downregulation of MMP-9 and intracellular adhesion molecule 1 expression, while also playing a crucial role in regulating apoptosis [82]. Ursolic acid, identified by bioactivity-guided fractionation of *Oldenlandia diffusa*, was shown to inhibit breast cancer metastasis by suppressing glycolytic metabolism through the activation of SP1/caveolin-1 signaling pathway [83]. Additionally, *Hedyotis diffusa Willd* (Rubiaceae), also known as *Oldenlandia diffusa* (*Willd*) *Roxb*., is widely recognized in Traditional Chinese medicine and is commonly used for the treatment of diseases with inflammation, such as hepatitis, appendicitis, and urethritis [84]. In conclusion, *Oldenlandia diffusa* (Willd.) Roxb. is a valuable Traditional Chinese medicine with notable antitumor and anti-inflammatory properties. In breast cancer cells, the *Oldenlandia diffusa* extract has been shown to inhibit proliferation and induce apoptosis by modulating key signaling pathways, and ursolic and oleanolic acids have been identified as bioactive compounds exerting these effects. Moreover, *Oldenlandia diffusa* suppresses breast cancer metastasis by inhibiting glycolytic metabolism and key metastatic signaling pathways, further reinforcing its therapeutic potential. Besides its antitumor effects, *Oldenlandia diffusa* has been traditionally used to treat inflammation-related diseases, underscoring its broad pharmacologic significance. Further research is warranted to elucidate these mechanisms and optimize the clinical utility of *Oldenlandia diffusa* and its bioactive components.

*Salvia miltiorrhiza*, a perennial herb belonging to the *Salvia* genus, has been widely used in Traditional Chinese medicine for centuries [85]. The anti-breast cancer components of *S. miltiorrhiza* are liposoluble tanshinones, such as dihydrotanshinone I, tanshinone I, tanshinone IIA, and cryptotanshinone, as well as water-soluble phenolic acids, such as rosmarinic acid and salvianolic acids A, B, and C (Table 5) [86]. Kim et al. demonstrated that the *Salvia miltiorrhiza* extract controlled metastasis in MCF-7 breast cancer cells through its ability to inhibit TPA-induced invasion [37]. Bioinformatics and systematic pharmacology studies revealed that the *Salvia miltiorrhiza*–ginseng combination significantly inhibited lung metastasis of breast cancer by increasing the expression of VEGF-A and MMP-9, which are involved in tumor angiogenesis and the formation of pre-metastatic niches, thereby improving the integrity of the tumor vascular basement membrane and inhibiting the EMT [87]. In summary, *Salvia miltiorrhiza* has significant therapeutic potential in breast cancer agent, owing to its bioactive components, including liposoluble tanshinones and water-soluble phenolic acids. Experimental studies show that the *Salvia miltiorrhiza* extract effectively inhibits the TPA-induced invasion of MCF-7 breast cancer cells. Furthermore, bioinformatic and pharmacologic analyses suggest that the *Salvia miltiorrhiza*–ginseng combination suppresses lung metastasis of breast cancer by modulating VEGF-A and MMP-9 expression, thereby enhancing vascular integrity and inhibiting the EMT. These findings highlight the therapeutic potential of *S. miltiorrhiza* in breast cancer treatment and warrant further investigation to elucidate the underlying mechanism and clinical applications.

## 3. Discussions

### 3.1. Variability in Experimental Design

Studies investigating the efficacy of herbal medicines, such as curcumin, in breast cancer treatment exhibit significant heterogeneity in their experimental designs [22]. Differences are evident in the choice of animal models, routes of administration including intravenous, intraperitoneal, intratumoral treatment durations, and the timing of intervention initiation relative to tumor induction or volume [88]. For instance, curcumin doses in preclinical studies range from 5 to 500 mg/kg, administered with varying frequencies and durations, leading to challenges in comparing results and drawing definitive conclusions [89,90].

### 3.2. Inconsistencies in Dosing and Bioavailability

Curcumin’s clinical application is hindered by its poor water solubility, rapid metabolism, and low bioavailability [91]. Oral administration often results in subtherapeutic plasma and tissue levels, limiting its efficacy [92,93]. To address this, various strategies have been employed, including the development of nanoparticle-based delivery systems, such as liposomes, polymeric nanoparticles, and micelles, which aim to enhance curcumin’s stability and bioavailability [94,95]. However, the lack of standardized dosing regimens and variability in formulation compositions across studies contribute to inconsistent outcomes and complicate the assessment of therapeutic potential on curcumin [96].

### 3.3. Variability in Outcomes

The outcomes reported in studies on herbal medicines for breast cancer treatment vary widely, influenced by differences in study design, dosing, and patient populations [97]. Some clinical trials have demonstrated promising results; for example, a randomized, double-blind, placebo-controlled study found that intravenous curcumin in combination with paclitaxel improved the objective response rate and physical performance in patients with advanced breast cancer [15,98]. Conversely, other studies have reported limited efficacy, potentially due to suboptimal dosing, poor bioavailability, or differences in patient characteristics [99,100]. These inconsistencies underscore the need for more rigorous and standardized research methodologies to accurately assess the therapeutic value of herbal medicines in breast cancer treatment.

Taken together, while herbal medicines like curcumin hold promise as adjuncts in breast cancer therapy, the current body of research is marked by significant variability and inconsistencies. Addressing these issues through standardized experimental designs, dosing regimens, and outcome measures is essential for advancing our understanding and application of these therapies in clinical settings.

## 4. Summary

Breast cancer is the most common cancer and the leading cause of cancer-related deaths among women worldwide. Significant advances in conventional treatments, such as surgery, chemotherapy, radiotherapy, hormone therapy, and targeted therapies, have improved outcomes; however, challenges such as drug resistance, recurrence, and adverse effects persist. Consequently, complementary approaches, including Traditional Chinese medicine and herbal therapies, are needed to improve treatment efficacy and quality of life.

**Figure 1:**
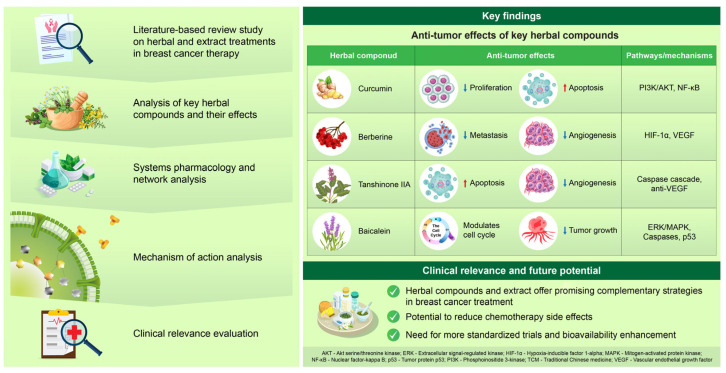
**Summary of key herbal and extract treatments for breast cancer.** The figure presents an overview of the anti-tumor effects and proposed mechanisms of action for four major herbal compounds: curcumin, berberine, tanshinone IIA, and baicalein. A literature-based review strategy was employed to analyze preclinical and clinical studies focusing on the active components of these herbs. The findings were integrated with systems pharmacology and network analysis to predict the effects on breast cancer. The compounds interact with critical molecular pathways, including PI3K/AKT, HIF-1α, VEGF, and caspase cascades, potentially enhancing the anti-tumor effects. Furthermore, there may be potential synergy with conventional therapies, offering the possibility to overcome drug resistance. While clinical data are limited, the preclinical evidence for the effectiveness of these compounds in treating ccRCC is promising. Further mechanistic and clinical validation is essential to confirm their therapeutic potential. ↑: Up-regulation; ↓: Down-regulation.

Herbal medicines are tailored through syndrome differentiation, aiming to restore balance using a variety of strategies, such as soothing the liver, regulating qi (i.e., vital energy in Traditional Chinese medicine), removing phlegm, activating blood, and nourishing yins. Numerous herbal compounds and formulations have demonstrated antitumor, anti-inflammatory, antioxidant, immunomodulatory, and proapoptotic effects in preclinical and clinical studies (Figure 1).

## 5. Conclusions

The integration of herbal medicines in breast cancer management represents a promising complementary approach to address the limitations of conventional therapies. Herbal compounds, such as curcumin, resveratrol, EGCG, WA, thymoquinone, baicalin, berberine, *Oldenlandia diffusa*, and *Salvia miltiorrhiza*, have significant antitumor effects, mediated through diverse mechanisms, including the induction of apoptosis, the suppression of metastasis, the modulation of immune responses, and the enhancement of chemotherapeutic efficacy. Preclinical as well as emerging clinical evidence underscores the therapeutic potential of these phytochemicals in overcoming drug resistance, improving quality of life, and reducing adverse effects. However, challenges such as standardization, bioavailability, and rigorous clinical validation remain. Continued interdisciplinary research that combines traditional knowledge with modern pharmacologic tools is essential for the safe and effective incorporation of herbal medicines into standard breast cancer treatment protocols.

## Figures and Tables

**Table 1:** Summary of therapeutic effects of curcumin in breast cancer.

Study Type	Findings	Mechanism/Outcome	References
Preclinical studies	Enhances effect of tamoxifen, reverses endocrine resistance	Inhibition of proliferation, promotion of apoptosis, targeting of survival pathways	[31]
Preclinical studies	Inhibits proliferation, induces apoptosis, suppresses metastasis	Modulation of multiple cancer-related molecular pathways	[14]
Preclinical study	Suppresses breast cancer cell proliferation	Downregulation of Flap endonuclease 1 via NRF2 signaling	[16]
Animal study	Intravenous curcumin inhibits tumor growth and metastasis in mice	Significant tumor suppression and antimetastatic activity	[17]
Clinical trial	Combination of curcumin with docetaxel suppresses breast cancer progression	Reduction in tumor marker levels	[19]
Clinical trial	Combination of curcumin with paclitaxel improves overall response rate and physical performance	Better treatment outcomes, reduced fatigue, and good tolerability	[15]
Pharmacokinetic study	Limitations in the clinical applicability of curcumin	Poor bioavailability, rapid metabolism, low water solubility	[22]
Drug delivery research	Development of advanced deliverysystems	Use of nanoparticles (polymeric nanoparticles, carbon nanotubes, liposomes) to enhance therapeutic efficacy	[23]

NRF2—nuclear factor erythroid 2-related factor 2.

**Table 2:** Antitumor activities of selected herbal compounds in breast cancer.

Herbal Compound	Clinical Trial Status	Key Findings	References
Curcumin (*Curcuma longa*)	Phase II randomized, double-blind, placebo-controlled trial (NCT03072992)	Intravenous curcumin (300 mg/week) combined with paclitaxel significantly improved objective response rate (ORR: 51% vs. 33%, *p* < 0.01) and physical performance in patients with advanced/metastatic breast cancer. Treatment was well-tolerated with reduced fatigue.	[15]
*Scutellaria baicalensis*	No registered clinical trials; preclinical studies available	Wogonin, a flavone from S. baicalensis, demonstrated induction of apoptosis and inhibition of proliferation in breast cancer cell lines. However, it also induced radioresistance in MCF-7 cells, indicating complex interactions.	[32]
*Oldenlandia diffusa*	Early genetic marker for progression	Aqueous extracts of O. diffusa induced apoptosis in breast cancer cell lines (MDA-MB-157 and 93B) via modulation of pro- and anti-apoptotic proteins, suggesting potential therapeutic effects.	[33]
*Salvia miltiorrhiza*	Observational study using Taiwan’s National Health Insurance Research Database (NHIRD)	Use of Danshen (S. miltiorrhiza) was associated with improved survival in breast cancer patients. Dihydroisotanshinone I, a compound from Danshen, induced ferroptosis and apoptosis in breast cancer cells in vitro and inhibited tumor growth in vivo.	[34]

**Table 3:** Anti-breast cancer properties of *Scutellaria baicalensis* and its bioactive compounds.

Compound/Extract	Active Components/Source	Mechanisms of Action	Breast Cancer Cell Line(s)	Key Outcomes	References
*Scutellaria baicalensis* (whole plant)	Traditional East Asian herb	Modulates transcription and kinase activity; alters phosphorylation in signaling pathways	Not specified	General anti-breast cancer effect via pathway modulation	[69,70]
Baicalin	Flavonoid from *Scutellaria baicalensis*	Inhibits β-catenin signaling, reverses EMT	MDA-MB-231	Suppresses metastasis in aggressive breast cancer	[72]
Baicalin + baicalein	Flavonoids from *Scutellaria baicalensis*	Activates caspases 9 and 3, downregulates BCL-2, upregulates BAX, p53; via ERK/p38 MAPK pathway	MCF-7	Synergistically enhance apoptosis	[73]
*Scutellaria baicalensis Georgi* extract	Ethanol extract	Increases ROS, activates MAPK/JNK, modulates BCL-2/BAX, activates caspases	Not specified	Inhibits proliferation and induces apoptosis; MAPK-dependent	[74]
*Scutellaria baicalensis* extract	Whole-plant extract	No effect with whole-plant extract, chemoprevention with specific fraction	MCF-7	Fractionated *Scutellaria baicalensis* extract shows selective antitumor effect	[75]

BAX—B-cell lymphoma 2-associated X protein; BCL-2—B-cell lymphoma 2; EMT—epithelial–mesenchymal transition; ERK—extracellular signal-regulated kinase; JNK—c-Jun N-terminal kinase; MAPK—mitogen-activated protein kinase; ROS—reactive oxygen species.

**Table 4:** Anti-breast cancer properties of *Oldenlandia diffusa (Willd.) Roxb.* and its bioactive compounds.

Compound/Extract	Active Components/Source	Mechanisms of Action	Breast Cancer Cell Line(s)	Key Outcomes	References
*Oldenlandia diffusa* extract	Whole-plant extract	Upregulates p53 via the ERα/SP1 pathway	Not specified	Inhibits proliferation, induces apoptosis	[81]
Ursolic acid and oleanolic acid (from *Oldenlandia diffusa* extract)	Bioactive triterpenoids	Induce p53, exert antiproliferative and proapoptotic effects	Not specified	Identified as active antitumor agents in *Oldenlandia diffusa* extract	[81]
*Oldenlandia diffusa*	Whole-plant extract	Inhibits p-ERK, p38, NF-κB; downregulates MMP-9 and ICAM-1	MCF-7	Reduces metastasis and invasion, promotes apoptosis	[82]
Ursolic acid (from *O. diffusa*)	Isolated by bioactivity-guided fractionation	Suppresses glycolytic metabolism via SP1/caveolin-1 signaling	Not specified	Inhibits metastasis	[83]
*Hedyotis diffusa* (synonym of *Oldenlandia diffusa*)	Traditional use	Used in treatment of diseases with inflammation (e.g., hepatitis, appendicitis)	Not specified	Anti-inflammatory and broad pharmacologic effects	[84]

Erα—estrogen receptor; ICAM-1—intracellular adhesion molecule 1; MMP-9—matrix metalloproteinase 9.

**Table 5:** Anti-breast cancer effects of *Salvia miltiorrhiza* and its active components.

Compound/Extract	Active Components	Mechanisms of Action	Breast Cancer Cell Line(s)	Key Outcomes	References
*Salvia miltiorrhiza* extract	Liposoluble tanshinones (e.g., dihydrotanshinone I, tanshinone I, tanshinone IIA, cryptotanshinone); phenolic acids (e.g., salvianolic acid A/B/C, rosmarinic acid)	Inhibits TPA-induced invasion	MCF-7	Reduces cell metastasis potential	[37]
*Salvia miltiorrhiza* extract–ginseng combination	Multi-herb formulation	Increases VEGF-A and MMP-9 expression, thereby enhancing vascular basement membrane integrity; inhibits EMT	Not specified	Inhibits lung metastasis, suppresses pre-metastatic niche formation	[87]

TPA—12-O-tetradecanoylphorbol-13-acetate; VEGF—vascular endothelial growth factor.

## Data Availability

Not applicable.

## Abbreviations

The following abbreviations are used in this manuscript:

ALDH	Aldehyde dehydrogenase
Bcl-2	B-cell lymphoma 2
BAX	B-cell lymphoma 2-associated X protein
BCL	B-cell lymphoma 2
CAF	Cancer-associated fibroblasts
CHM	Chinese herbal medicine
CHOP	C-EBP homologous protein
CHP	Chinese herbal prescription
CMH	Chinese medicinal herbs
Elk1	ETS-like transcription factor 1
DRP1	Dynamin-related protein 1
EGCG	Epigallocatechin gallate
EMT	Epithelial–mesenchymal transition
ER	Estrogen receptor
Erα	Estrogen receptor alpha
Fen1	Flap Endonuclease 1
IBC	Inflammatory breast cancer
IL-12	Interleukin 12
JNK	c-Jun N-terminal kinase
MAPK	Mitogen-activated protein kinase
MCF-7	Michigan Cancer Foundation-7 (human breast cancer cell line)
MDA-MB-231	A human breast cancer cell line
MMP	Matrix metalloproteinase
NF-κB	Nuclear factor kappa-light-chain-enhancer of activated B cells
NRF2	Nuclear factor erythroid 2-related factor 2
ODE	*Oldenlandia diffusa* extract
OPA	Optic atrophy protein
ORR	Overall response rate
POLD1	DNA polymerase delta catalytic subunit gene
p-eIF2α	Phosphorylated eukaryotic initiation factor 2 alpha
p-PERK	Phosphorylated PKR-like ER kinase
ERK	Phosphorylated extracellular signal-Regulated kinase
PMNs	Pre-Metastatic niches
Qi	Vital energy (in Traditional Chinese medicine)
ROS	Reactive oxygen species
RSK	Ribosomal S6 kinase
RSV	Resveratrol
SBGE	*Scutellaria baicalensis* Georgi extract
SbE	*Scutellaria baicalensis* extract
SG	*Salvia miltiorrhiza*–Ginseng
SGLXD	Shu-Gan-Liang-Xue Decoction
STS	Steroid sulfatase
TCA	Tricarboxylic acid
TCM	Traditional Chinese medicine
VEGF-A	Vascular endothelial growth factor A
